# Concurrent coronary and extra-coronary spontaneous artery dissection in Ehlers–Danlos syndrome: multimodal imaging insights

**DOI:** 10.1093/ehjimp/qyae101

**Published:** 2024-09-28

**Authors:** Marta Zielonka, Tania Ramírez-Martínez, Ramon Bascompte Claret, Isabel Hernández-Martín, Kristian Rivera

**Affiliations:** Department of Cardiology, Arnau de Vilanova University Hospital, Av. Rovira Roure 80, 25198 Lleida, Spain; Department of Cardiology, Arnau de Vilanova University Hospital, Av. Rovira Roure 80, 25198 Lleida, Spain; Department of Cardiology, Arnau de Vilanova University Hospital, Av. Rovira Roure 80, 25198 Lleida, Spain; Department of Cardiology, Arnau de Vilanova University Hospital, Av. Rovira Roure 80, 25198 Lleida, Spain; Department of Cardiology, Arnau de Vilanova University Hospital, Av. Rovira Roure 80, 25198 Lleida, Spain; Grup de Fisiologia i Patologia Cardíaca, Institut de Recerca Biomèdica de Lleida, Fundació Dr. Pifarré, IRBLleida, Lleida, Spain

**Keywords:** Ehlers–Danlos syndrome, peripheral arterial dissection, spontaneous coronary artery dissection, multimodal imaging

**Figure:**
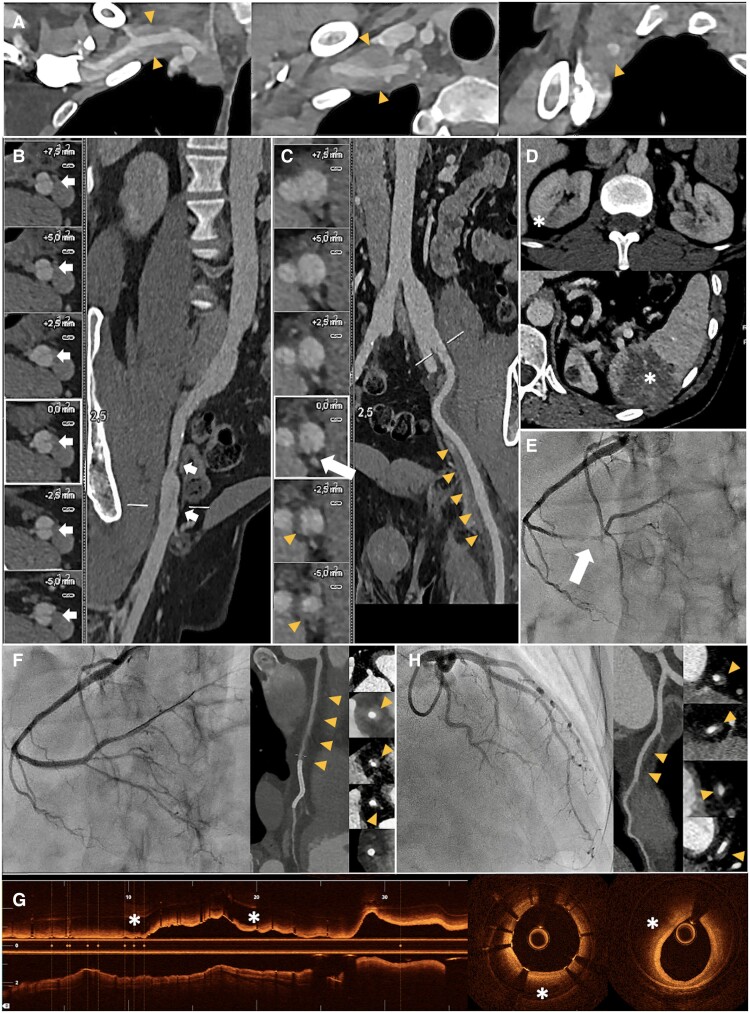


A 41-year-old male presented with abdominal pain following a prolonged flight. He was diagnosed with Ehlers–Danlos syndrome (EDS) by the COL3A1 gene mutation 8 years earlier, after a spontaneous dissection of the right common iliac artery during the course of a half-marathon. In the current admission, computed tomography (CT) angiography revealed a mural haematoma in the right subclavian artery *(Panel A)* and a known chronic dissection flap in the right external iliac artery *(Panel B)*. A flap was identified in the left external iliac artery, associated with concentric mural thickening suggestive of a haematoma *(Panel C, arrow and triangles, respectively)*. Bilateral kidney ischaemic lesions with repletion defects in the posterior branches of both renal arteries and a splenic ischaemic lesion *(Panel D)* were also noted. Conservative treatment was indicated.

Twenty-four hours after admission, he experienced a non-ST elevation myocardial infarction. Emergency coronary angiography (CAG) revealed a type 2 spontaneous coronary artery dissection in the distal segment of the right coronary artery (RCA) *(Panel E)*, with impaired coronary flow. A sirolimus-eluting stent was successfully implanted, restoring distal coronary flow *(Panel F)*. Optical coherence tomography showed a large intramural haematoma that extends from the mid-segment of the stent to the entire proximal RCA *(Panel G)*. A subsequent coronary CT scan highlighted a mural haematoma in the proximal and mid-RCA with a patent stent and an additional haematoma in the mid-distal anterior descending artery, which had not been visualized on CAG *(Panel H)*. Multimodal imaging was crucial in this rare presentation of EDS, helping to identify concurrent, symptomatic, and clinically inadvertent coronary and peripheral arterial dissections and guide therapeutic decisions.

## Data Availability

No new data were generated or analysed in support of this research.

## Lead author biography

Kristian Rivera graduated from the University of Colima, Mexico, and later specialized in internal medicine at the Western Medical Center in Guadalajara, Mexico, and cardiology at the National Institute of Cardiology Ignacio Chavez in Mexico City. He completed a fellowship at Bellvitge University Hospital in Barcelona, Spain, and is currently an interventional cardiologist at Arnau de Vilanova University Hospital in Lleida, Spain, where he is completing his PhD studies.

